# Risk factors for injuries in female soldiers: a systematic review

**DOI:** 10.1186/s13102-022-00443-z

**Published:** 2022-03-29

**Authors:** Ben Schram, Elisa Canetti, Robin Orr, Rodney Pope

**Affiliations:** 1 Tactical Research Unit, Bond Institute of Health and Sport, Bond University, 2 Promethean Way, Robina, QLD 4229 Australia; 2 School of Community Health, Charles Sturt University, Albury, NSW Australia

**Keywords:** Tactical, Military, Women, Injury

## Abstract

**Background:**

Female soldiers form an integral part of any modern defence force. Previous reports have highlighted that female soldiers report injuries at higher rates than male personnel. One possible reason for this is an actual difference in underlying injury rates, purported to be due to several factors, including levels of fitness. The aim of this review was to determine risk factors for injuries in female soldiers.

**Methods:**

A systematic search was conducted for studies which reported on risk factors for injuries in female soldiers. Databases searched included PUBMED, CINAHL and Medline through OVID. Eligible studies were rated for their methodological quality using the Critical Appraisal Skills Program (CASP) tools and data were extracted and synthesized using a critical narrative approach.

**Results:**

A total of 18 articles were included in this review which reported on 18 risk factors for injury. Smoking, previous injury, no history of deployment, heavy occupational tasks, lower levels of aerobic fitness and lower number of push-up repetitions appear to be risk factors for injuries in female soldiers. Age, height, body fat, high or low BMI and body mass do not appear to be consistent risk factors for injury in female soldiers and there appears to be minimal evidence for current levels of activity, sit-up ability, and other assessments of strength, power, speed, or movement being associated with injury risk. Additionally, neither flexibility nor previous levels of activity appear to be associated with injury risk in female soldiers.

**Conclusion:**

Strategies to improve aerobic fitness and upper limb endurance, reduce smoking, and optimise rehabilitation from injuries and risk management for heavy occupational tasks need to be developed for female soldiers. Such strategies are also likely to reduce risks for male soldiers.

## Background

With more direct combat roles being made available [1], female soldiers continue to play an integral part in a modern military [2]. Ensuring female personnel can perform at an optimal level, free of the burden of injury is therefore imperative to maintaining combat readiness and effectiveness [3]. Musculoskeletal injuries are detrimental to operational readiness [4], causing a higher rate of hospitalization than direct combat related injuries in deployed personnel [5–7]. Reduction of injuries in military organisations is therefore considered a force multiplier [8].

Female soldiers have been found to experience injuries at a higher rate, both in training [9, 10] and during operations [11, 12] when compared to their male colleagues, although this sex-based difference does not hold true across all military contexts and in some contexts male personnel have been observed to have higher injury rates than female personnel [13, 14]. There is also evidence that female soldiers may sustain injuries to different body sites when compared to male soldiers [15–17] and exhibit some differences in risk factors for injury, due to anthropometric, biomechanical, and anatomical differences. In basic training contexts where male and female recruits train together, despite males experiencing greater external training loads as measured by total distance covered, female soldiers tended to have a greater internal training load as measured by heart rate and ratings of perceived exertion and report more muscle soreness and fatigue [18].

Given that, historically, injury reduction programs have been designed primarily around male soldiers, as they comprise the greatest proportion of army personnel, these programs may not be optimally managing risks of injuries in females. Given the reported differences between male and female soldiers, the aim of this review was to identify, analyse, and synthesize findings from studies which have reported on risk factors for injuries in female soldiers, to inform targeted injury reduction programs.

## Methods

This review was registered as part of a broader research project with Prospero (CRD42020170003). To source articles relevant to this review, dedicated search terms were developed after a preliminary rapid search. The databases PubMed, CINAHL, and Medline through OVID were searched systematically using the themes of female, military (and army), and injury, or derivatives thereof. Articles found through reference lists or though the authors knowledge, but not identified by the databases, were also considered for inclusion. An example of the search terms used can be found in Table 1.

**Table 1:** Example of the search terms used in Pubmed

Theme 1	Theme 2	Theme 3
female[Title/Abstract] OR women[Title/Abstract] OR woman[Title/Abstract]	injur*[Title/Abstract]	defence[Title/Abstract] OR defense[Title/Abstract] OR military[Title/Abstract] OR army[Title/Abstract] OR tactical[Title/Abstract] OR recruit[Title/Abstract] OR soldier[Title/Abstract] OR cadet[Title/Abstract] OR trainee[Title/Abstract]

### Inclusion and exclusion criteria

Studies were included if they: (a) reported on risk factors for injuries sustained by female army personnel, (b) were original, peer reviewed research, (c) were available in full text, and (d) were written in English, or translatable to English by the reviewers. Due to the differences in basic training, entry standards, and occupational demands between services, the focus of this review was narrowed to army personnel only. To gain an understanding of any differences in injury experiences throughout an army career, articles which reported on either training or operational army soldiers were included. Articles were excluded if: (a) they did not report on risk factors separately for female personnel, (b) focused on a specific injury type so that a generalisation for all injuries could not be drawn, (c) pertained to a more severe level of injury only (e.g., hospitalisation or medical discharge), (d) were from a specialised occupation within the Army (e.g. Military Police), or (e) were inclusive of part time or reserve personnel or military services other than army and data for full-time soldiers were not separable for extraction. If articles reported on injury risk of combined cohorts of part time and full-time personnel who undertook the same training program on a full-time, short-term basis, they were retained, and all personnel were treated as if they were full time personnel. Studies which included enlisted part time personnel were excluded due to the risk of confounding arising from the inability to control for the other occupational and recreational activities undertaken for many more hours than full-time personnel would have had available for such pursuits.

Search results were imported into Endnote software (Endnote X9, version X9.3.3, Clarivate Analytics, Philadelphia, United States), where duplicates were removed, and articles were screened by title and abstract by two reviewers to assess potential eligibility for inclusion. The inclusion and exclusion criteria were then applied to the remaining articles through detailed review of full texts of the articles by two reviewers, with any disagreements settled by discussion with a third reviewer. The results of the search, screening and selection processes were recorded in a PRISMA flow chart [19].

Key data from the included studies were then extracted, tabulated, and synthesised. Data of interest included the authors and year of publication, the population size and environment, the risk factors examined, and indicators of the levels of association between specific risk factors and injury risk (for example, odds ratios (OR), relative risks (RR), hazard ratios (HR) or incidence rate ratios (IRR)).

The methodological quality of each included article was appraised using the Critical Appraisal Skills Program (CASP) [20] tool for cohort studies or the AXIS tool for cross sectional studies [21]. The CASP has 12 questions, with a maximum possible score of 12, with both questions 5 and 6 containing two sections, but questions 7 and 8 not being scored, due to their subjectivity. The AXIS has 20 questions with a total possible score of 20. The first 11 questions of the AXIS tool relate to objectives and methods, the next seven to the study’s findings and the final two to ethical considerations. The raw scores from each tool were consolidated into one score, converted to a percentage and given an accompanying methodological quality rating, whereby scores < 45.4% were deemed to indicate poor quality, scores 45.4%-61.0% fair quality and scores > 61.0% good quality [22]. A score was assigned to appraisals in this review because it provides a crude but useful indicator of overall methodological quality to compliment the narrative description of each study. The methodological quality score was included in the data table to allow for the data from each study to be considered in the context of the methodological quality of the respective study.

A critical narrative approach was taken to the qualitative synthesis of findings from the included studies. Meta-analysis was not conducted due to heterogeneity in study designs and methods, injury definitions, outcome measures, and risk factors explored.

## Results

**Fig. 1:**
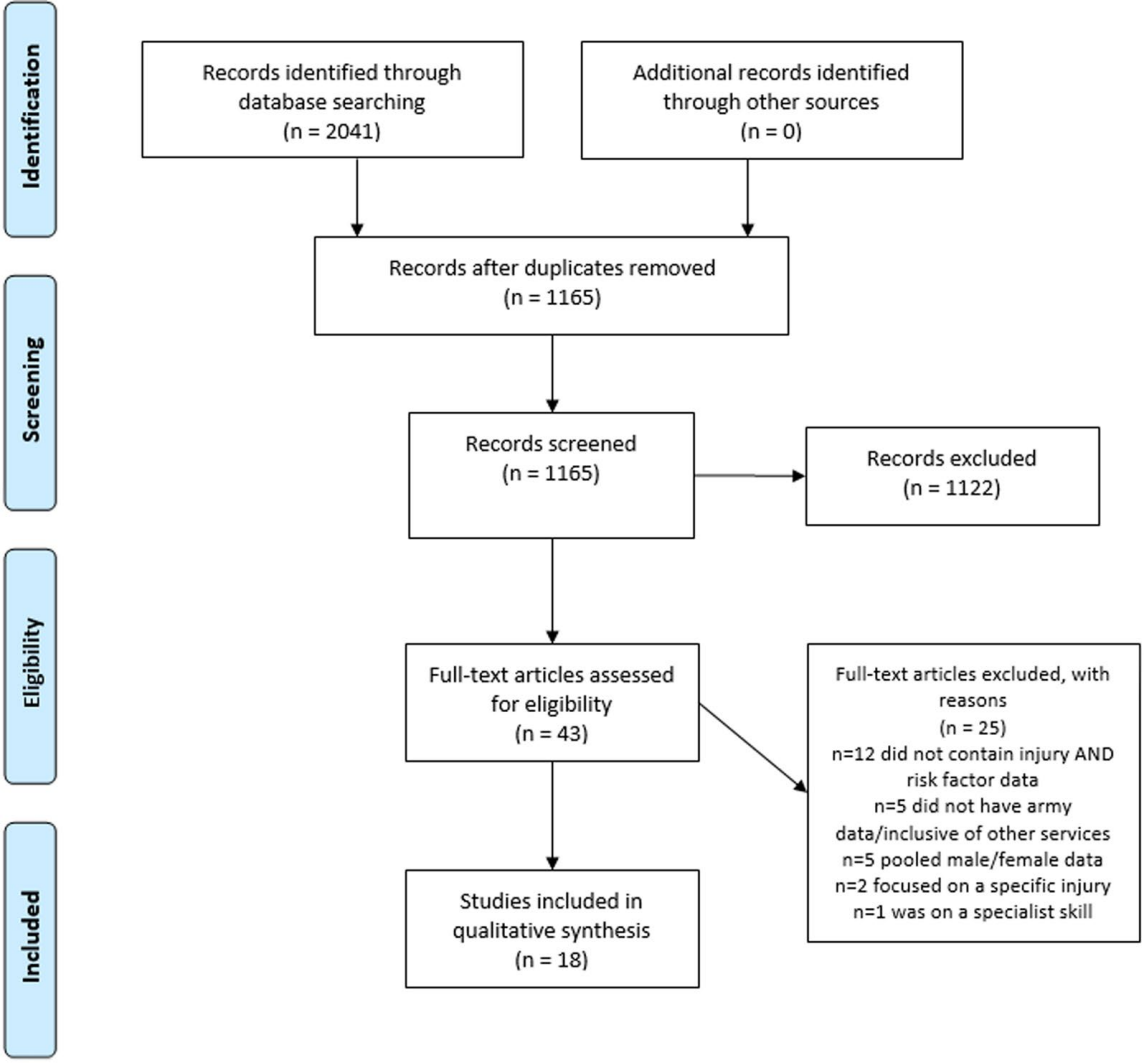
PRISMA flow chart [19] showing the screening and selection process

From an initial 1165 articles screened after duplicates were removed, 18 studies were eligible and included in the review (Fig. 1). There were 15 cohort studies [1–3, 9, 23–33] and three cross-sectional studies [34–36]. Methodological quality overall was considered ‘good’ (80%), with cohort studies tending to score higher (82%), than cross-sectional studies (68%). Seven studies assessed injury risk in basic training [16, 23, 25–29], three studies during Advanced Individual Training (AIT) [24, 35, 36], three studies in enlisted personnel [1, 3, 34], three studies during deployments [2, 32, 33], one study during officer training [9], and one during the first 183 days of service [31]. Sixteen of the included studies were conducted in the United States military [1, 2, 2, 3, 9, 23, 24, 26–29, 31, 36], one in the Israel Defense Forces [30], and one in the British Army [25]. A total of 17 potential risk factors for injury were investigated, ranging from demographic and anthropometric factors, such as age, height, and weight, to physical performance measures, such as aerobic fitness, or muscle endurance, and to historical factors, such as smoking or injury history, previous activity levels, or deployment history. Factors which were similar, such as running a variety of distances, were grouped for comparison in the synthesis below.

### Age

In total, eight studies assessed the influence of age on injury risk in female soldiers [1, 3, 24, 28, 29, 34–36], and found conflicting results, with four studies finding older age was a risk factor for injury, one finding younger age was a risk factor, and three finding no relationship between age and injury risk. In basic training, female soldiers in the US Army were found to be at a greater injury risk in the age brackets of 25–29.9 years (and over 30 years when compared to those aged 17–19.9 years [29]. Conversely, Knapik et al. [28] found that age was not associated with injury risk in female recruits undertaking Army basic training when comparing 17–20-year-old recruits with 20–25- and 35–35-year-old recruits. Two [24, 36] out of three studies performed in the AIT context found that older females were at an increased risk of injury. Women attending AIT in the US Army were significantly more likely to report an injury they had suffered during recruit training which they perceived would affect their current training if they were aged 20–24 years or over 30 years[24] when compared to 17–19 year olds and in another study there was similarly a significantly greater injury incidence in those aged 20–25 years [36] than in younger soldiers. Contrasting the aforementioned results, in a population of females attending Ordinance AIT in the US Military, age was not found to be significantly associated with the risk of a time loss injury in older groups when compared to those aged 17–19 years (reference group) [35].

Three studies were conducted on enlisted personnel after completion of both basic training and AIT [1, 3, 34], with these studies finding conflicting results. Age was not found to be associated with injury risk for enlisted females within the US Army in a study by Rappole et al. [1], while Anderson et al. [34] found that younger Army soldiers who were women aged 22 to 26 years were more likely to be injured than both those aged 27 to 30 years and those over 31 years. However, another study of all newly qualified US Army soldiers by Bedno et al. [3] found older females were at a greater risk of lower limb injury and that, when compared to those who were aged 17–23 years, each increasing age group was at a progressively higher risk of suffering a lower limb injury (Table 2).

**Table 2:** Risk factors for injuries in female military personnel

References study type	Population	Risk factor	Key findings		Score* (%)
1. Altarac et al. [23] cohort study	915 female US Army recruits in 8-week basic training. Smoking history captured by self-reported survey prior to training, injuries collected via medical record review	Smoking in 1 month prior to enlisting	Smoking vs not smoking		92
			**Any injury**	**aOR 1.61 [95% CI = 1.19–2.17]**	
			Traumatic	aOR 1.05 [95% CI = 0.67–1.64]	
			Overuse	aOR = 1.71 [95% CI = 1.26–2.31]	
			** ≥ 1 days lost injury**	**aOR = 1.44 [95% CI = 1.02–2.02]**	
			** ≥ 6 days lost**	**aOR = 1.75 [95% CI = 1.21–2.51]**	
2. Anderson et al. [34] cross-sectional study	363 female enlisted US Army soldiers from two light infantry brigades. Data collected via self-reported survey over 12 months	Age	22–26 years (57% injured), 27–30 years (43% injured), ≥ 31 years (48% injured)		60
		BF %	≤ 19.28%	REFERENCE	
			19.29–23.37%	RR = 0.74 [95% CI = 0.30–1.80]	
			≥ 23.38%	RR = 0.88 [95% CI = 0.53–1.47]	
		APFT push-ups	≥ 72 reps	REFERENCE	
			58–71 reps	RR = 1.05 [95% CI = 0.32–3.48]	
			≤ 57 reps	RR = 1.30 [95% CI = 0.44–3.82]	
		Sit-ups	≥ 74 reps	REFERENCE	
			63-73reps	RR = 1.09 [95% CI = 0.78–1.54]	
			**≤ 62 reps**	**RR 1.35 [95% CI = 1.01–1.80] p = 0.03**	
		2-mile run time	≤ 14.13 min	REFERENCE	
			14.14–15.67 min	RR = 0.92 [95% CI = 0.35–2.41]	
			≥ 15.68 min	RR = 1.32 [95% CI = 0.61–2.85]	
3. Bedno et al. [3] cohort study	All 37,413 new enlisted female US Army soldiers from Jan 2011–Jan 2014 (lower extremity ICD = 9 musculoskeletal injuries captured from inpatient or outpatient visits)	Age	**17–23 years**	**REF**	83
			**24–28 years**	**OR = 1.05 [1.01–1.10] p = 0.008**	
			**29–35 years**	**OR = 1.18 [1.11–1.26] p < 0.001**	
			**≥ 36 years**	**OR = 1.47 [1.27–1.69] p < 0.001**	
		BMI	Normal	REFERENCE	
			Underweight	OR = 1.16 [0.99–1.36] p = 0.07	
			Overweight	OR = 1.04 [1.00–1.08] p = 0.09	
			Obese	OR = 1.03 [0.90–1.18] p = 0.68	
		APFT	> 270	REFERENCE	
			**240–269**	**OR = 1.11 [95% CI = 1.03–1.20] p = 0.006**	
			**215–239**	**OR = 1.17 [95% CI = 1.09–1.27] p < 0.001**	
			** < 215**	**OR = 1.45 [95% CI = 1.35–1.57] p < 0.001**	
		Current smoking (Y/N)	**(Y)**	**OR = 1.30 [1.23–1.36]**	
		Deployment	none	REFERENCE	
			**1**	**OR = 0.65 [95% CI = 0.58–0.72] p < 0.001**	
			2	OR 0.73 [95% CI = 0.43–1.25] p = 0.258	
4. Bijur et al. [9] cohort study	85 female West Point Cadets. Data collected via medical records	Height	N/S		92
		Mean Run time per mile	7.2 min 42.1 injuries /100 cadets, 8.1 min 66.7 injuries /100 cadets, 8.7 min 76.2 injuries /100 cadets, 9.8 min 126.3 injuries/100 cadets		
5. Grier et al. [24] cohort study	3856 women prior to ordinance AIT after recently finishing BCT. All data collected via self-reported survey	Age	17–19 years	REFERENCE	70
			**20–24 years**	**OR = 1.29 [95% CI = 1.07–1.56]**	
			25–29years	OR = 1.29 [95% CI = 0.96–1.74]	
			**≥ 30 years**	**OR = 2.02 [95% CI = 1.43–2.87]**	
		Smoking	Non-smokers	REFERENCE	
			Occasional	OR = 1.01 [95% CI = 0.74–1.39] p = 0.95	
			Frequent	OR = 0.96 [95% CI = 0.80–1.15] p = 0.65	
		Number of Cigarettes	None	REFERENCE	
			≤ 10	OR = 1.04 [95% CI = 0.80–1.34] p = 0.77	
			20-Oct	OR = 0.91 [95% CI = 0.71–1.18] p = 0.48	
			> 20	OR = 0.89 [95% CI = 0.61–1.30] p = 0.55	
6. Grier et al. [35] cross-sectional study	498 Women at US Army Ordinance School. Physical performance data collected via military records; injuries collected via self-reported survey	Age	17–19 years	REFERENCE	83
			20–24 years	HR = 0.89 [95% CI = 0.68–1.16) p = 0.39	
			25+ years	HR = 1.18 [95% CI = 0.86–1.63] p = 0.32	
		Smoking	Non-smokers	REFERENCE	
			Occasional	HR = 1.08 [95% CI = 0.67–1.73] p = 0.77	
			Frequent	HR = 1.27 [95% CI = 0.98–1.63] p = 0.07	
		Number of Cigarettes	None	REFERENCE	
			≤ 10	HR = 1.03 [95% CI = 0.71–1.49] p = 0.89	
			20-Oct	HR = 1.37 [95% CI = 0.95–1.97] p = 0.09	
			** > 20**	**HR = 1.71 [95% CI = 1.12–2.59] p = 0.01**	
		Injury (Y/N)	**Y**	**HR = 1.67 [95% CI = 1.21–2.30] p < 0.01**	
		Push-ups	**0–23 reps**	**HR = 1.47 [95% CI = 1.03–2.09] p = 0.03**	
			**24–30 reps**	**HR = 1.44 [95% CI = 1.02–2.04] p = 0.04**	
			31–36 reps	HR = 1.04 [95% CI = 0.71–1.53] p = 0.84	
			37 + reps	REFERENCE	
		Sit-ups	0–53 reps	HR = 1.28 [95% CI = 0.91–1.76] p = 0.16	
			54–60 reps	HR = 1.17 [95% CI = 0.83–1.66] p = 0.36	
			61–67 reps	HR = 1.04 [95% CI = 0.73–1.48] p = 0.82	
			68 + reps	REFERENCE	
		2 Mile Run	0–17.00 min	REFERENCE	
			**17.01–18.08 min**	**HR = 1.46 [95% CI = 1.02–2.08] p = 0.04**	
			18.09–19.38 min	HR = 1.27 [95% CI = 0.88–1.83] p = 0.21	
			**19.39 + mins**	**HR = 2.04 [95% CI = 1.45–2.88] p < 0.01**	
7. Heller et al. [25] cohort study	227 British Army basic training recruits. Run times collected via military records, injuries collected via medical records	1.5 mile run time	Mean injury free time = 12min13sec, Mean Injured time = 12min43sec, Every 10 s increase in time = 8.3% greater injury risk		75
8. Henderson et al. [36] cross-sectional study	287 US Combat Medic Trainees. Previous injury, smoking and activity collected via self-reported survey; all other data collected via medical records	Age	< 20 years Injury Incidence 31.2%	REFERENCE	75
			20–25 years Injury Incidence 21.3%	aOR = 0.7 [95% CI = 0.4–1.3]	
			**> 25 years Injury Incidence 52.9%**	**aOR = 3.5 [95% CI = 1.5–8.1]**	
		Previous Injury	Y Injury incidence 29.5%, N Injury Incidence 37.4%		
		BMI	17.5–21.0 kg/m^2^ Injury Incidence 22.1%		
			22.0–23.5 kg/m^2^ Injury Incidence 29.0%		
			23.6–25.3 kg/m^2^ Injury Incidence 27.9%		
			25.4–31.2 kg/m^2^ Injury Incidence 36.8%		
			n/s p = 0.305		
		Body Mass	46-57 kg Injury Incidence 22.4%	REFERENCE	
			58-63 kg Injury Incidence 30.9%	aOR = 1.5 [95% CI = 0.7–2.3]	
			64-68 kg Injury Incidence 22.2%	aOR = 1.1 [95% CI = 0.5–2.5]	
			**69-95 kg Injury Incidence 41.8%**	**aOR = 2.4 [95% CI = 1.1–5.0]**	
		Smoking (Y/N)			
		Activity Prior to BCT			
		Frequency of Activity prior to BCT	Yes—Injury Incidence 23.6%, No—Injury Incidence 31.8% (n/s p = 0.190)		
			More than most—Injury Incidence 41.9%, Somewhat more than most—Injury Incidence 25.6%, as active as most—Injury Incidence 29.5%, Less active than most—Injury Incidence 25.0% (p = 0.243)		
			0/week—Injury Incidence 36.4%		
			1–2/week– Injury Incidence 25.3%		
			3–4/week– Injury Incidence 31.0%		
			5–7/week– Injury Incidence 30.9% (p = 0.623)		
9. Jones et al. [26] cohort study	186 women Army trainees during basic training. Previous activity self-reported via survey, all other data via medical records,	Height	**Shorter 25% women at greater risk than taller 75%**	**RR 1.7 [95% CI = 1.2–2.4] p = 0.02**	83
		Body Fat %	N/S		
		Body mass	N/S		
		BMI	N/S		
		Push-ups	N/S		
		Sit-ups	N/S		
		Self-reported previous activity	N/S		
		Run Time	Q1	RR = 1.16 [95% CI = 0.5–2.7]	
			Q2	RR = 1.0	
			Q3	**RR = 2.40 [95% CI = 1.2–4.8] p = 0.028**	
			Q4	RR = 2.18 [95% CI = 1.1–5.0] p = 0.063	
		**Slow vs Fast Runners**	**Any injury**	**RR = 1.69 [95% CI = 1.2–2.4] p = 0.004**	
			**Lower body injury**	**RR = 1.78 [95% CI = 1.2–2.6] p = 0.004**	
			**Time loss injury**	**RR = 2.12 [95% CI = 1.2–3.7] p = 0.0007**	
			**Stress Fracture**	**RR = 2.54 [95% CI = 1.0–6.6] p = 0.05**	
10. Jones et al. [27] cohort study	41 727 women in US Army Basic Training. Data collected via military database; injuries collected via surveillance system	2-mile Run time	Q1 (Fastest) injury risk = 26.5%	REFERENCE	83
			**Q2 Injury risk = 35%**	**RR = 1.3 [95% CI = 1.3–1.4]**	
			**Q3 Injury risk = 39.3%**	**RR = 1.5 [95% CI = 1.4–1.5]**	
			**Q4 Injury risk = 44.6%**	**RR = 1.7 [95% CI = 1.6–1.8]**	
			**Q5 (Slowest) Injury risk = 56.0%**	**RR = 2.1 [95% CI = 2.0–2.2]**	
		BMI	**Q1 (Lowest) Injury risk = 41.9%**	**RR = 1.1 [95% CI = 1.02–1.1]**	
			Q2 Injury risk = 39.1%	RR = 1.0 [95% CI = 0.9–1.0]	
			Q3 Injury risk = 39.5%	REFERENCE	
			Q4 Injury risk = 39.6%	RR = 1.0 [95% CI = 0.9–1.1]	
			**Q5 (Highest) Injury risk = 41.2%**	**RR = 1.04 [95% CI = 1.01–1.08]**	
		Slow run time and low BMI	**Injury risk 63.1%**	**RR = 2.6 [95% CI = 2.3–2.8], p < 0.00001**	
		Push-ups	**Low vs High 48.8% to 31.6%**	**RR = 1.5 [95% CI = 1.49–1.61] p < 0.00001**	
		Low BMI & Low Push-ups	**Injury risk 50%**	**RR = 1.7 [95% CI = 1.6–1.9]**	
11. Knapik et al. [28] cohort study	474 women in US Army Basic Training. Smoking and physical activity history self-reported, all other data collected via medical records	Age	17–20 years	REFERENCE	92
			20–25 years	RR = 1.0 [95% CI = 0.8–1.3]	
			25–35 years	RR = 1.3 [95% CI = 0.9–1.9]	
		Height	58–62in	REFERENCE	
			63–64in	RR = 1.0 [95% CI = 0.7–1.4] p = 0.89	
			65–66in	RR = 1.3 [95% CI = 0.9–1.9] p = 0.22	
			67–74in	RR = 1.0 [95% CI = 0.7–0.4] p = 0.85	
		Mass	90–119lbs	REFERENCE	
			120–134lbs	RR = 1.0 [95% CI = 0.7–1.4] p = 0.93	
			135–150lbs	RR = 1.0 [95% CI = 0.7–1.5] p = 0.84	
			151–239lbs	RR = 1.1 [95% CI = 0.8–1.6] p = 0.47	
		BMI	15.81–20.54 m/kg^2^	REFERENCE	
			20.55–22.98 m/kg^2^	RR = 1.3[95% CI = 0.9–1.9] p = 0.15	
			22.99–25.01 m/kg^2^	RR = 0.9 [95% CI = 0.7–1.4] p = 0.78	
			25.02–33.21 m/kg^2^	RR = 1.3 [95% CI = 0.9–1.9] p = 0.10	
		**3.2 km Run time**	13.00–19.48 min	REFERENCE	
			19.49–21.65 min	RR = 1.5 [95% CI = 1.0–2.3] p = 0.06	
			**21.66–23.48 min**	**RR = 1.6 [95% CI = 1.0–2.3] p = 0.04**	
			**23.49–28.68 min**	**RR = 1.9 [95% CI = 1.2–2.8] p < 0.01**	
		**Push-ups**	**0–2 reps**	**RR = 1.6 [95% CI = 1.1–2.5] p = 0.02**	
			**3–5 reps**	**RR = 1.6 [95% CI = 1.1–2.3] p = 0.02**	
			**6–13 reps**	**RR = 1.6 [95% CI = 1.1–2.4] p = 0.02**	
			14–50 reps	REFERENCE	
		Sit-ups	0–22 reps	RR = 1.3 [95% CI = 0.9–2.0] p = 0.14	
			23–33 reps	RR = 1.2 [95% CI = 0.8–1.8] p = 0.29	
			34–44 reps	RR = 1.1 [95% CI = 0.7–1.6] p = 0.66	
			45–80 reps	REFERENCE	
		**Smoking (Y/N)**	**Y**	**RR = 2.0 [95% CI = 12–3.5] p = 0.01**	
			** > 20 Cigarettes/day**	**RR = 1.4 [95% CI = 1.9–10.0] p < 0.01**	
		**VO**_**2max**_	**29.9–37.0 ml/kg/min**	**RR = 2.8 [95% CI = 1.4–5.6] p < 0.01**	
12. Knapik et al. [29] cohort Study	451 women in US Army Basic Training. Smoking, injury, and activity history collected via self-reported survey; injuries collected via surveillance system	**Age**	17.0–19.9 years	REFERENCE	100
			20.0–24.9 years	HR = 1.02 [95% CI = 0.85–1.23] p = 0.84	
			**25.0–29.9 years**	**HR = 1.30 [95% CI = 1.01–1.66] p = 0.04**	
			**≥ 30 years**	**HR = 1.43 [95% CI = 1.12–1.84] p < 0.01**	
		**Previous injury (Y/N)**	**(Y)**	**HR = 1.41 [95% CI = 1.13–1.75] p < 0.01**	
		BMI	15.20–22.12 kg/m^2^	REFERENCE	
			21.30–23.80 kg/m^2^	HR = 0.89 [95% CI = 0.71–1.11] p = 0.30	
			23.81–25.97 kg/m^2^	HR = 0.91 [95% CI = 0.73–1.13] p = 0.40	
			25.98–34.02 kg/m^2^	HR = 0.89 [95% CI = 0.71–1.11] p = 0.28	
		**Push-ups**	**0–4 reps**	**HR = 1.92 [95% CI = 1.41–2.59] p < 0.01**	
			5–13 reps	HR = 1.36 [95% CI = 0.99–1.86] p = 0.06	
			14–22 reps	HR = 1.20 [95% CI = 0.87–1.65] p = 0.27	
			23–62 reps	REFERENCE	
		**Sit-ups**	**0–20 reps**	**HR = 1.75 [95% CI = 1.29–2.37] p < 0.01**	
			21–33 reps	HR = 1.34 [95% CI = 0.98–1.83] p = 0.07	
			34–46 reps	HR = 1.10 [95% CI = 0.79–1.51] p = 0.58	
			47–89 reps	REFERENCE	
		**2 Mile Run**	12.3–19.4 min	REFERENCE	
			19.5–22.1 min	HR = 0.99 [95% CI = 0.71–1.38] p = 0.94	
			22.2–24.7 min	HR = 1.14 [95% CI = 0.82–1.59] p = 0.43	
			**24.8–31.3 min**	**HR = 2.18 [95% CI = 1.60–2.98] p < 0.01**	
		**Smoking**	**0**	REFERENCE	
			**1–9/day**	**HR = 1.44 [95% CI = 1.19–1.73] p < 0.01**	
			**10–19/day**	**HR = 1.47 [95% CI = 1.17–1.89] p < 0.01**	
			**≥ 20/day**	**HR = 1.90 [95% CI = 1.34–2.68] p < 0.01**	
		**Frequency of ex before BCT**	**≤ 1/week**	**HR 1.41 [95% CI = 1.09–1.82p p < 0.01**	
		**Frequency of running before BCT**	**≤ 1/week**	**HR = 1.62 [95% CI = 1.16–2.27] p < 0.01**	
13. Kodesh et al. [30] cohort study	158 females on IDF Combat Fitness Instructor Course. All data collected via medical records	Power Performance including 10 m sprint, single leg triple hop drop jump, CMJ	All power tests n/s except, **L triple hop (cm) 418 (254–559) vs 446.5 (199–584) p = 0.029, R triple hop distance (cm) 434 (287–536) vs 460 (263–546) p = 0.047)**		67
		Body Fat %	**BF% 23.7 (20.5–29.2) vs 22.5 (14.9–31.5) p = 0.047**		
		BMI	BMI 21.14 [18.06–25.79) injured vs 20.70 [16.16–32.03] not injured		
		FMS	FMS n/s		
		2 km Run	**258 (578–776) sec vs 640 (488–804) sec p = 0.044**		
14. Krauss et al. [31] cohort study	1900 US Army recruits during first 183 days of service from six locations in the US. Data collected via military and medical records	Fit vs unfit	**Non stress fracture**	**IRR = 1.32 [95% CI = 1.14–1.53]**	75
			**Stress fracture**	**IRR = 1.62 [95% CI = 1.19–2.21]**	
			**Non stress fracture**	**IRR = 1.27 [95% CI = 1.07–1.50]**	
		Fit (high % BF vs low % BF)	Stress fracture	IRR = 0.79 [95% CI = 0.49–1.28]	
15. Rappole et al. [1] cohort study	369 US female Army Soldiers from Combat Arms, Combat Support and Combat Service Support. Injury data collected via medical records, other data via self-reported survey	Age	n/s		67
		2-mile run	n/s		
		Push-ups	n/s		
		Sit-ups	n/s (at 0.05)		
		Unit PT resistance training	**≥ 1/week**	**OR 1.96 [95% CI = 1.20–3.21] p < 0.01**	
		Personal PT run distance	> 1 mile	OR 1.57 [95% CI = 0.98–2.52] p = 0.06	
		Personal PT interval freq	**None/ < 1/week**	**OR 1.64 [95% CI = 1.00–2.71] p = 0.05**	
16. Roy et al. [2] cohort study	625 US Army female soldiers within three brigade combat teams Injuries collected via self-reported survey	Deployment	0	RR = 1.48 [**95% CI = **1.02–1.71]	75
			1	RR = 1.22 [**95% CI = **0.84–1.78]	
			≥ 2	REFERENCE	
		Injury History (Y/N)	**(Y)**	**RR = 2.6 [95% CI = 2**.06–3.28]	
		2-mile Run time	≤ 17 min	REFERENCE	
			**17.01–18.0**	**RR = 1.71 [95% CI = 1.07–2.73]**	
			> 18 min	RR = 1.41 [95% CI = 0.90–2.19]	
		Unit Runs/week	**0**	**RR = 1.526 [95% CI = 1.07–2.19]**	
		Pers. PT/week	0	REFERENCE	
			**2-Jan**	**RR = 1.42 [95% CI = 1.08–1.87]**	
			≥ 3	RR = 1.31 [95% CI = 0.94–1.83]	
		APFT score	**< 220**	**RR = 1.74 [95% CI = 1.01–3.02]**	
			**220–249**	**RR = 2.01 [95% CI = 1.19–3.38]**	
			**250–289**	**RR = 1.7 [95% CI = 1.02–2.86]**	
			≥ 290	REFERENCE	
17. Roy et al. [32] cohort study	57 female soldiers from US infantry or support battalions on deployment in Afghanistan in 2012. Data collected by self-reported survey	Work type	**Physically demanding**	**RR = 6.0 [95% CI = 1.50–23.99]**	67
		Miles walked/day	**> 4**	**RR = 3.0 [95% CI = 1.52–5.93]**	
		Avg worn load	**> 30 lb**	**RR = 2.44 [95% CI = 1.7–4.36]**	
		Weight of avg lifted object	**> 50 lb**	**RR = 2.3 [95% CI = 1.19–4.45]**	
18. Roy et al. [33] cohort study	160 female soldiers from three brigade combat teams deployed to Afghanistan for nine months. Data collected via self-reported survey	Wearing load	**> 10% body weight**	**RR = 2.0 [95% CI = 1.31–3.06]**	100
			**Heaviest load > 15%**	**RR = 5.83 [95% CI = 1.51–22.50]**	
		Wearing armour	**1–4 h**	**RR = 1.62 [95% CI = 1.002–2.62]**	
			**> 4 h**	**RR = 1.84 [95% CI = 1.03–3.27]**	
		Wearing backpack	**Y**	**RR = 1.85 [95% CI = 1.23–2.80]**	
		Occupational tasks	**Lifting > 22.68 kg**	**RR = 1.96 [95% CI = 1.08–2.97]**	
			**Lifting objects 1–2 × day**	**RR = 1.73 [95% CI = 1.002–2.97]**	
			**Carrying objects > 7.62 m**	**RR = 2.01 [95% CI = 1.19–3.42]**	
		Y balance score	**< 95.23**	**RR = 1.71 [95% CI = 1.13–2.60]**	

Bold indicates significant risk factors

RR = Relative Risk, OR = Odds Ratio, IRR = Incident Rate Ratio, HR = Hazard Ratio, 95% CI = 95% Confidence Interval, n/s = not significant, in = inches

^*^Methodological Quality Score

Three of four studies within basic training and one of two articles in AIT showed that higher age was associated with an increased injury risk. The one study in Ordinance AIT which showed no difference between age groups in injury risk may be due to the age groups selected in the analysis. The upper age bracket of > 25 years included personnel comparably younger than the upper age group of > 30 years used in basic training studies and may have therefore reduced the magnitude of the association observed between age group and injury risk. The conflicting results across these studies regarding the relationship between age and injury risk in female soldiers do not seem to form any clear patterns, and indicate it is currently unclear whether younger or older age is a risk factor for injury and that other factors may be more important predictors of injury risk.

### Body mass index

Body Mass Index (BMI) and its relationship to injury risk were considered in seven studies [1, 3, 26–29, 36]. BMI was not found to be significantly associated with injury risk in female personnel in all but one [27] of the four studies conducted during basic training [26–29], in the one study conducted during AIT [36], and in the two studies involving enlisted personnel [1, 3]. BMI was not a significant predictor of lower limb musculoskeletal injury in the study by Bedno et al. [3], when comparing risk in enlisted female soldiers who were considered to be underweight (< 18 kg/m^2^), overweight (25–29.9 kg/m^2^) or obese (≥ 30 kg/m^2^) to risk in those who were considered to be of normal weight (18–24.9 kg/m^2^). In contrast, in the same study, BMI was found to be a significant predictor amongst male personnel, with underweight, overweight, and obese male soldiers more likely to suffer a lower limb musculoskeletal injury when compared to those considered to be of normal weight.

In contrast, in a large study of 41,727 female recruits undertaking basic training, Jones et al. [27] did find a significant bimodal relationship, with low and high BMI associated with increased injury risk. Both the low BMI group of < 20.7 kg/m^2^ and high group of > 25.6 kg/m^2^ were found to be at an increased risk of injury when compared to those considered to be in the ‘normal’ range for BMI. This finding suggests that perhaps BMI has not been identified as a significant risk factor in studies with smaller sample sizes due to lower statistical power of those studies to detect such a relationship. In addition, Jones et al. [27] proposed that lower BMI may be more problematic than a higher BMI due to its association with lower muscle mass. The authors of the study also suggested that BMI interacted with fitness, which was deemed to be more critical, and that those with a high BMI would be at less risk if they had adequate aerobic fitness. Overall, the volume of evidence suggests that BMI is not a strong and consistent risk factor for injury in female soldiers and that other factors may be more important in predicting injury risk.

### Body fat

Body fat percentage is thought to be a more accurate representation of body composition than BMI [37] and was investigated in five studies [1, 26, 28, 30, 34], with four studies [1, 26, 28, 34] finding no relationship between body fat percentages and injury risk. Body fat percentage was found not to be a predictor of injury risk during basic training for female soldiers in the US Army in two studies [26, 28], and was also not found to be associated with injury risk in a study of enlisted female US Army personnel [1]. Despite a study by Anderson et al., [34] finding no difference in injury rates between tertiles of body fat percentage in female soldiers from the US Army, in male soldiers, those in the middle and highest tertile were injured more commonly than those in the lowest tertile (≤ 29.05%), suggesting while body fat percentage might be a risk factor for male soldiers, it is not an important risk factor in female soldiers. In contrast to this finding, Kodesh et al. [30] found a significant difference in body fat levels between those female soldiers who were injured when compared to those who were not injured during a three month Combat Fitness Instructor Course in the IDF. The female soldiers in that study were much leaner (22.5%) than those in other studies (25.2 ± 9.36% [26] to 31.7 ± 5.3% [34]), and this may have affected their findings. Nevertheless, the volume of evidence suggests that body fat percentage, in general, is not a strong risk factor for injury in female soldiers.

### Body mass

Body mass was examined as a potential risk factor for female soldiers in three studies [26, 28, 36], with only one finding a significant relationship between body mass and injury risk [36]. Female personnel who weighed between 69 and 95 kg had a greater incidence of injury during Combat Medic AIT when compared to those weighing less [36]. A regression analysis found an OR for injury of 2.4 [95% CI = 1.1–5.0] for those of that weight category when compared to lighter female soldiers [36]. Noting the broad confidence interval for this OR in the study by Henderson [36], and the non-significance of body weight as a risk factor for injury in female recruits in the other two studies of body mass, it would appear body mass is unlikely to be a strong risk factor for injury in female soldiers.

### Height

Only one study [26] of four that investigated body height as a potential risk factor [9, 26, 28, 36] found a relationship between height and injury risk in female soldiers. Only the study by Jones et al. [26] found that the females in the quartile of shorter stature (~ 164 cm) were at a greater risk of injury during basic training than the taller 75%. Height, therefore, does not appear to be a strong risk factor for injury in female soldiers.

### Smoking

Five [3, 23, 28, 29, 35] out of seven of the included studies that investigated smoking as a potential risk factor [3, 23, 24, 28, 29, 35, 36] found that smoking was a risk factor for injury among female soldiers. Smoking history was collected via survey or questionnaire in all studies except the study by Bedno et al. [3], who utilised reports from periodic health assessments. Altarac et al. [23] found that female soldiers who had smoked prior to enlisting in the US Army presented with an increased risk of injury overall, overuse and more severe injuries, but not traumatic injuries. Female recruits in US Army basic training who smoked > 20 cigarettes [28] or as few as 1–9 cigarettes per day [29] were found to be at a significantly greater risk of injury than female recruits who did not smoke. In a similar manner, Bedno et al. [3] found that female US Army soldiers who were currently serving and smoking were at a greater risk of lower limb injury than serving female soldiers who were not smoking.

Grier et al. [35] reported that, overall, females who were both occasional (smoked on less than 20 out of the previous 30 days) and frequent smokers (smoked on 20 or more of the previous 30 days) prior to initial training were not at a significantly increased risk of time loss injury when compared to those who did not smoke. However, when the female soldiers were stratified by the number of cigarettes smoked, a significantly greater risk was evident for those who smoked 20 cigarettes or more per day in the 30 days prior to basic training when compared to those who smoked fewer than 20 cigarettes per day. In contrast, Grier et al. [24], in their earlier work published in 2010, found that among female soldiers, neither occasional smokers nor frequent smokers were at a significantly greater risk of injury when compared to those who did not smoke, and that comparisons of groups based on number of cigarettes smoked per day did not suggest increases in smoking were associated with an increased risk of injury. Likewise, Henderson et al. [36] found no evidence that female soldiers who reported they were current smokers were at an increased risk of injury during combat medic AIT when compared to those who were not smokers. The volume of evidence nevertheless suggests that smoking is a significant risk factor for injury in female army personnel.

### Previous injury

Four studies examined the association between history of previous injury and future injury risk in female soldiers [2, 29, 35, 36], with three [2, 29, 35] showing a significant relationship. Female personnel who had suffered a previous self-reported injury were found to be at an increased risk of subsequent injury during US Army Basic Training [29]. Likewise, those who had reported a previous injury were at a greater risk of suffering a ‘time loss’ injury than those who did not report a previous injury during US Army Ordinance school training [35]. Enlisted female US Army soldiers were shown to be an increased risk of injury if they had a history of injury [2]. Conversely, work by Henderson et al. [36] found that an injury suffered in basic training did not lead to a higher injury incidence in AIT training for combat medics.

### Current physical activity

There are conflicting results regarding the association between reported current level of physical activity performed by female soldiers and injury risk. Rappole et al. [1] found that female soldiers of light infantry brigades who were doing more unit physical training (PT), but less personal running and interval training, were at an increased risk of injury. The female enlisted US Army soldiers who were doing unit PT more than once a week were reported to be at a greater risk of injury than those who were doing fewer sessions [1]. However, those who were *not* doing personal running of at least one mile per week or personal interval training at least once per week were found to be at increased risk of injury when compared to those who were doing one of these types of personal training, though the differences were of marginal statistical significance. Conversely, in the study by Roy et al. [2], enlisted female soldiers in the US Army were found to be at an increased risk of injury if they did not do any unit runs each week, when compared to those who did one to two runs per week [2]. In addition, those female soldiers who did one to two personal resistance training sessions per week were reported to be at a greater injury risk than those who did none.

### Previous physical activity

Four studies assessed the relationship between previous levels of physical activity performed by female soldiers prior to basic training [26, 28, 29] and AIT [36] and injury risk. Only one of the studies conducted during basic training [29] showed an association between self-reported previous activity and injury risk; those who reported that they participated in sport or exercise less than once per week prior to basic combat training were found to be at a greater risk of injury than those who reported greater than five episodes of sport or exercise per week. A similar result was seen for self-reported running or jogging in the same study, with those reporting a history of less than one session per week being at a greater risk of injury than those two reported five or more sessions per week [29]. Conversely, the self-reported amount or duration of physical activity prior to enlistment was not found to be associated with injury incidence in female soldiers undertaking combat medic training [36], or in female recruits undertaking basic training in two other studies [26, 28].

### Deployment

The relationship between deployment history and injury risk was investigated in two studies [2, 3], while individual risk factors for injury during operations in Afghanistan were the focus of two other studies [33, 38]. Female soldiers of US Army units who had not been deployed were found to have higher injury rates than those who had been deployed at least twice [2]. A similar finding was reported by Bedno et al. [3], whereby those who had been on one deployment were less likely to be injured than those who had not been deployed.

### Heavy occupational tasks

Whilst on deployment, there have been several risk factors reported as increasing the likelihood of injury among female soldiers. Self-reported physically demanding work, walking more than four miles per day, wearing loads greater than 30 pounds, carrying loads for more than 25 feet, lifting objects to waist height or lower, wearing armour for more than an hour a day, wearing a backpack, and lifting an average weight of greater than 50 pounds were all found to be associated with an increased risk of injury in female soldiers deployed in Afghanistan, in two studies by Roy et al. [33, 38].

### APFT

Two studies [2, 3], both involving enlisted, female army personnel, investigated the relationship between injury risk and overall score on the Army Physical Fitness Test (APFT), comprised of a 2-mile run, push-ups, and sit-ups in 2 min. Female soldiers who had a score lower than 270 points on the APFT were found to be at an increased risk of lower limb injury, with a gradual increase in risk for those in lower scoring categories [3]. Roy et al. [2] found an increased risk even in those scoring up to 290 points, with 290 points being used as a threshold level beyond which risk of injury was lower than that observed in those scoring below that score (Table 2).

### Push-ups

Seven studies investigated the relationships between push-up performance and injury risk in female personnel. In trainees, three [27–29] of four studies [26–29] reported a relationship between low push-up performance and injury risk during basic training while the remaining study failed to find a significant association.

In addition, the by Jones et al. [27] found low BMI combined with low push-up ability was associated with an increased injury risk when compared to those with normal BMI in the highest push-up quintile.

A study by Grier et al. [35], involving 498 female soldiers, investigated push-up performance and injury risk during US Army AIT at the Ordinance School and found those who could perform more than 37 push-ups were at a decreased risk of time loss injury when compared to those who could perform less [35]. Conversely, both studies of enlisted female Army personnel serving after completion of initial training failed to find any association between push-up performance and injury risk [1, 34]. No difference was found in injury rates between any tertiles of push-ups within female enlisted US Army soldiers; this contrasts with the finding for male soldiers, in whom risk of injury was found to be increased for male soldiers who performed less than 62 repetitions [34]. The single study conducted in advanced training also found a relationship, despite the two conducted in active-duty female personnel not finding a significant relationship. The average number of repetitions performed in the basic training environment by female personnel is substantially lower (n = 10.6–12.4 repetitions [27, 29]) than that in the female active-duty population (n = 37 repetitions [1, 35]), which may explain this finding. The volume of evidence suggests that low push-up performance may be a risk factor for injury within female soldiers during basic training but potentially not for female soldiers serving after completion of basic training.

### Sit-ups

Four studies examined sit-up performance in two minutes as a potential risk factor for injuries in female soldiers during basic training [26–29], one during AIT [35], and two in enlisted soldiers [1, 34]. Only the study in 2009 by Knapik et al. [29] found that the number of sit-ups in 2 min was a risk factor for injury, with risk increased in those female personnel who could only perform 0–20 repetitions, when compared to those who could perform more than 47 repetitions.

Later in the training progression of a soldier, the maximal number of sit-up repetitions was not found to be a risk factor for injury in female soldiers undertaking Ordnance School AIT in the US Army.

Female enlisted US Army personnel who performed in the bottom two thirds of sit-up repetitions for the APFT were found to be at an increased risk of injury when compared to those in the upper third, however APFT sit-up repetitions did not feature in a multivariate regression model for prediction of injury in that population [1]. Enlisted female soldiers who could not perform more than 62 sit-ups were found to be at a greater risk of injury than those who could perform more than 62, and a similar risk of injury was found in enlisted men who were unable to complete that number Overall, the volume of evidence suggests sit-up performance may be a weak predictor of injury risk in female soldiers, but the findings are inconsistent.

### Strength, power and speed assessments

Kodesh et al. [30] conducted a barrage of power and speed assessments on female soldiers completing a combat fitness instructors’ course in the Israel Defense Forces (IDF). Neither the 10 m sprint time nor any parameters measured for the drop jump and the counter movement jump were found to be significantly associated with injury risk. The single leg triple hop distance was found to be significantly associated with injury risk. Knapik et al. [28] also assessed a variety of strength and power measurements in female recruits undertaking basic training, including incremental dynamic lift strength, upper and lower body strength, upright pull static strength, and a vertical jump assessment, and found no relationship between any measurement and injury risk. Overall, the evidence at this stage does not support or suggests only weak associations between low levels of strength, power or speed and injury risk in female military personnel.

### Flexibility

Knapik et al. [28] assessed flexibility in females undertaking basic training with the sit-and-reach test and found no significant difference in injury risk for those who scored less, or more than the referent group of 32-39 cm. In contrast, the male trainees did show a relationship between their sit-and-reach performance and injury risk, with a bimodal curve evident in which those who were most and least flexible were at greater risk of injury.

### Aerobic fitness

Aerobic fitness, as measured by runs of varying distances, including 3.2 km/2mile [2, 27–29, 35], 2.4 km/1.5 mile [25], 2 km [30], or 1 mile [26], a 5 min step test [31], or average time per mile [9], was found to be related to injury risk in eleven studies that investigated this potential risk factor for injury, and at all stages of a female soldiers career. The one exception was the study by Rappole et al. [1], in which the run distance was 2 miles, and completed by 369 female soldiers.

Krauss et al. [31] explored the interaction between body fat, aerobic fitness, and injury risk in female US Army trainees. They found that those who were deemed to be unfit, as measured by a five-minute step test, were more prone to both non stress fracture injury and stress fracture injury than those who were fit. Those who were fit but exceeded body fat limits had an increased risk of non-stress fracture injury but appeared to be less prone to stress fracture when compared to females who were both fit and of optimal body fat composition. Those who met the body fat limits but were unfit tended to suffer more stress fractures. Conversely, Rappole et al. [1] did not find 2-mile run times to feature in the multivariate model which was predictive of lower extremity, training related injury among enlisted women (n = 369) in the US Army, suggesting that in enlisted women serving in the army, aerobic fitness levels may not be strong predictors of injury risk and other factors may be more important in predicting injury risk.

### Movement assessments (FMS & Y Balance)

One study examined the relationships between Functional Movement Screen (FMS) results and injury during a combat fitness instructors course within the Israel Defense Forces (IDF) [30]. No significant difference was found in FMS scores between those injured and not injured. In attempting to find an optimal cut-off score in the FMS which could be used for prediction of injury risk, Kodesh et al. [30] found that with a score of 12, they only achieved 24% sensitivity and 83% specificity, while a score of 14 led to 42% sensitivity and 63% specificity. Their regression model for prediction of injury risk based on FMS scores was not statistically significant, leading the authors to suggest that the FMS was not an effective tool in predicting injury risk in female soldiers in the IDF.

Single leg balance ability and its relationship to injury amongst female soldiers deployed in Afghanistan was assessed in one study [33]. Roy et al. [33] found that those female soldiers who had a composite score of ≤ 95.23 in the Y Balance assessment were at a significantly greater risk of injury than those who scored above this number. There were no other studies which reported associations between injury risk and results of any other movement assessments.

## Discussion

The aim of this review was to identify, analyze, and synthesize studies which reported on injury risk factors specific to female soldiers, to inform targeted injury reduction programs. Most of the included studies (16/18) were from the US Army, with a variety of environments studied, from basic training, to AIT, and to enlisted and deployed personnel. Overall, smoking, previous injury, history of no deployment, heavy occupational tasks while on deployment, low aerobic fitness and poor push-up performance appear to be associated with heightened injury risk in female military personnel. Age, height, body mass, body fat, BMI and flexibility do not appear to be related, or appear to be inconsistently related, to injury risk. Minimal research has been performed on flexibility, current levels of activity and assessments of strength, power, speed, and movement as potential risk factors in female personnel. Given that some known risk factors for injury in males, such as older age, BMI, body fat percentage, flexibility, and previous activity levels [3, 30, 35, 37], are not evident in female personnel, the reasons for these apparent sex differences warrant further investigation.

### Age

Age has been inconsistently associated with injury risk in female military personnel. The study by Henderson et al. [36] used the same age bracket in Combat Medic AIT, however, due to the less physically arduous nature of that AIT training, it is difficult to make direct comparisons between it and other AIT training. This supposition is supported by Tomes et al. [39], who noted that the associations between injury risk and fitness factors in tactical populations were influenced by the training being undertaken by personnel.

Of the two studies conducted in enlisted personnel who were not undertaking a training program, one found that younger age was a risk factor for injury, and one found no difference in risk across age groups. This latter finding might be explained in part by the increase in age that typically accompanies higher ranks and the decrease in physical tasks undertaken by those of a higher rank, which has been demonstrated in previous studies [40]. It has been reported that for each individual increase in rank for female soldiers, the injury risk decreases 14% [2]. When soldiers perform the same tasks, such as during basic training and AIT, injury risk is often higher in older personnel [36]. Older age is typically associated with declines in cardiorespiratory fitness, which in itself is associated with an increased risk of injury [41], and may explain the increased injury risk during training [42]. In contrast, changes in physically demanding tasks with increased rank may explain the lack of associations between age and injury in serving populations. Across all studies, a phenomenon similar to the healthy worker effect may also influence the results, whereby those who have been injured may have been discharged from the military, leaving only those who are more injury resilient to form older study populations; further affecting findings regarding age as a risk factor for injury [43]. Age therefore is not a consistent risk factor for injury in female soldiers.

### BMI, body fat and body mass

Only one of the six studies which reported on BMI found an association with injury risk in female soldiers. The higher levels of BMI may be associated with an increased amount of lean muscle mass and BMI’s inability to offer insight into body composition make it problematic for drawing meaningful conclusions regarding underlying mechanisms for any association with injury risk.

Krauss et al. [31] found female soldiers with high levels of body fat suffered more non-stress fracture injuries and were at a lower risk of stress fractures than those with lower levels of body fat. The results of that study may not be generalizable, however, as it was inclusive of individuals who exceeded the body fat limits for entry to the army and who were granted a waiver for a short period of time [31]. The four other studies did not find body fat to be a risk factor for injury in female soldiers. Body fat was associated with injury risk in males in two studies [26, 34], and it is not clear why this risk factor is different between males and females. Although errors arising from body fat being estimated in most instances with various equations or from four-site skinfold assessments may contribute to variation in study findings, body fat was not related to injury in the study by Knapik et al. [28], when using DEXA to determine body composition.

Only one of three studies found a relationship between body mass and injury risk in female soldiers [36]. The authors of that study proposed that this finding was due to greater body forces arising from the extra mass, potentially making those with greater levels of body fat more susceptible to injury [36]. Conversely, those with greater mass, BMI or body fat may be more musculoskeletally resilient, provided they possess adequate aerobic fitness [34]. BMI, body fat or body mass therefore are not consistent risk factors for injury in female soldiers.

### Height

Of four studies [9, 26, 28, 36], three failed to find any relationship between height and female soldier injury risk [9, 28, 36]. There was only one study, the earliest of the four studies, conducted in 1993, which found height to be related to injury risk [26]. Injuries in females who were shorter were linked to overstriding when marching at the rear of formations in a study in 2000 [44], and subsequently adequate steps may have been put in place to ensure shorter individuals set the pace of march at the front of a formation. This may explain the lack of association between height and injury risk in female soldiers since the early 1990’s. Conversely, there may be a progressive increase in mean heights of the population, which has been reported in United States military cohorts and might reduce the impact of this risk factor [45]. The findings of this review suggest that height is not a risk factor for injury in female soldiers.

### Smoking

Smoking appears to be a risk factor for injury amongst female soldiers, with most studies finding a significant association between smoking and injury risk, particularly for overuse type injuries [23]. Smoking is reported to affect bone mineral density, have effects on fibroblasts—affecting both the healing of injuries and the tissue repair process—and contribute to overall injury risk [23, 35].

The lack of association in the earlier study by Grier et al. [24] may be explained by the self-reporting of injuries and the question posed to participants of whether they thought their injuries may affect their training. Attendees to AIT may be reluctant to disclose an injury or they might perceive that their injury may not affect their training, given they may not know much about its composition or the variation in physicality and length of AIT courses. Likewise, recruits are not able to smoke during either basic training or AIT, which may have led to a ‘wash out’ of the detrimental effects of smoking over this period [36]. A smoking history is therefore considered a risk factor for injury in female soldiers.

### Previous Injury

An injury history appears to be associated with risk of future injury in female soldiers, with three of four included studies showing a relationship with injury risk. The one study which did not show a significant relationship between previous injury and injury risk during AIT may have had this finding due to the self-reporting of injury, the short duration of the study, or the reported lower physical intensity of combat medic training when compared to other forms of military training [35]. Injuries during basic training are reported to be suffered at a much higher rate than at other times during a military career [46], and this could potentially lead to a career full of injuries if these initial injuries predispose female soldiers to subsequent injuries [47]. In military populations, previous injuries both at the same body location and at adjacent locations are reported to lead to a ten-fold increase in future injury risk [48–50]. The same relationship is seen in sporting contexts [51], and with sporting participation being a notable cause of injury in both male and female military personnel post-basic-training, reduction of sporting injuries should also be a priority [52]. Emphasis should therefore be placed on ensuring injuries, when they do occur, are fully rehabilitated prior to female (and male) soldiers returning to training or active duty, to minimise the chance of further injury. The results of the included studies suggest that a previous injury is a risk factor for future injury in female soldiers.

### Current and previous activity

Of the three included studies which assessed the level of activity performed by female soldiers prior to basic training, two of them asked participants to rate themselves compared to others. It is unclear whether this perception of one’s own level of activity biases the result, as Knapik et al. [29] found that there were no significant increases in injury risk for those who rated themselves at any level compared to others. However, research does indicate that military personnel are generally reliable when self-rating their fitness [53]. When questions pertaining to frequency of exercise or sport, and history and frequency of running were posed, significant increases in injury risk were found in those who reported doing less [29]. Self-reported level of physical activity prior to joining the army was not found to be associated with injury risk in female soldiers during subsequent AIT training, proposed to be due to normalization of physical activity levels through the 8 weeks of basic training [36]. There may have also been a basement effect, whereby recruits were required to be fit enough to pass the entry tests, thereby having the requisite level of fitness to minimise injury risk.

Aerobic fitness, muscular strength and endurance are occupationally relevant to female soldiers and play a role in reducing the risk of injury [54]. Studies included in this review found that enlisted females who did not perform their own running or interval training and participated in unit runs were found to be at an increased risk of injury [1, 2]. Conversely, both unit [1] and personal [2] resistance training were found to increase risk of injury for female enlisted soldiers. Resistance training is important for female soldiers, as it increases lean muscle mass and strength [1]. Roy et al. [2] found that those who were doing 1–2 session of individual resistance training were more commonly injured than those doing none or at least three sessions; leading the authors to recommend further research with a larger sample size (the sample size in their study was 625 women). It has been proposed that perhaps more instruction in resistance training is needed for female soldiers [1], however, it is unclear if it is simply the addition of resistance training to the already physically demanding nature of deployment which contributes to overall injury risk, as opposed to the resistance training itself—a supposition supported by the work of Goodall et al., [55] who found that the inclusion of balance and agility training in addition to current training actually increased injury risk rather than decreasing it. Unit resistance training, in the study by Rappole et al., [1] may not have been individualized, and therefore contributed to, as opposed to decreased, overall injury risk. This highlights once again, the importance of ability-based training within military personnel. Overall, the results suggest that there is only minimal evidence for current levels of activity, while previous levels of activity do not appear to be associated with injury risk in female soldiers.

### Deployment

The reason for deployment being protective against injury in female soldiers may relate to several factors. Soldiers must reach a certain level of fitness to be deployed, and therefore those who do not meet these standards or are injured, will likely not be deployed. Thus, a pseudo healthy worker effect may be present. Research has shown that while personnel are on deployment, 2-mile run times decreased by 50 s, due to an increase in personal running [56]. This may be due to the ability of deployed personnel to self-pace their training, as formal PT is atypical on deployment, which has been shown to improve fitness and decrease injury risk [57]. A decrease in unit PT, combat training and sport may also explain some of the decline in injury rates, given that these are leading causes of injury [2, 58]. Not having been deployed may therefore be a risk factor for injury in female soldiers.

### Heavy occupational tasks

Whilst on deployment, there are a range of physical and mental stresses which a soldier is exposed to due to a lack of resources, austere environmental conditions, and the potential for hostile contacts [56]. Despite great efforts directed at reducing combat-based injuries and fatalities, training related injuries and other non-combat injuries are typically responsible for a higher number of hospitalisations than combat related injuries [5–7]. Female military personnel appear to be at an increased risk of injury while on deployment when lifting, carrying, and wearing load [32, 33, 38, 59]. It is an occupational requirement for soldiers working in combat zones to carry load, including body armour, which for females, can be plagued by issues with equipment fitting and conforming to the female body [60], given that it is typically designed for males. Despite reports of significantly lower absolute loads being carried by female soldiers, their relative loads were the same as for male soldiers in a study conducted prior to female soldiers entering combat trades [61]. As such, the removal of combat operation restrictions for female soldiers may see female soldiers carrying similar absolute loads but heavier relative loads, a supposition supported by research in law enforcement [62]. Furthermore, female soldiers have been shown to be working at a higher work effort in carrying equivalent absolute load to male soldiers, due to differences in absolute fitness and body composition [15]. The potential increases in absolute load, leading to a higher relative load, and resulting requirement for female soldiers to work at a higher work effort carrying these loads could be anticipated to increase injury risk during combat-specific training and on deployments. Both carrying and lifting these heavy loads on deployment have been highlighted as a risk factor for injury in female personnel [32, 33]. Other deployment related risk factors for injury include wearing equipment including backpacks and armour for longer durations, and work which is considered to be heavy and physically demanding [32, 33].

### APFT

Poor scores on the APFT were associated with injury risk for female soldiers [2, 3]. It is unclear however, how much of the overall score is influenced by the individual elements of push-ups, sit-ups, and the 2-mile run, and so each of these elements is discussed in detail below.

### Push-ups

Three out of four studies conducted in the basic training environment found a relationship between a low level of push-ups performed in 2 min and injury risk in female trainees. Push-up performance may also be a proxy measure for general fitness [63], and exposure to physical training within the military may be adequate to increase fitness such that it is no longer a risk factor for injury. Furthermore, it may be that tasks performed in the military context are not overly reliant on upper limb endurance [27], and that perhaps measures of lower limb endurance may be more relevant, as lower limb endurance is more related to military tasks [64]. A lower number of push-up repetitions appears to be a risk factor for injury in female soldiers.

### Sit-ups

Overall, there were variable results with respect to sit-up performance and injury risk for female soldiers, with just three of seven articles showing a relationship. Only one study in basic training, but both articles in active service personnel found a relationship between sit-up performance and injury risk. In line with push-up performance, sit-up performance may be an indicator of global fitness [63], with those who are more active being more proficient at this assessment. Sit-up performance, as a measure of trunk muscle endurance, has shown little correlation with common military tasks [64], with some questioning the appropriateness of the sit-up assessment within this environment [27]. There only appears to be minimal evidence for the association between poor sit-up performance and injury risk in female soldiers.

### Strength, speed, and power

The inclusion of strength, speed, or power assessments in military contexts has increased, with both the vertical jump and grip strength assessments now being added to the Canadian military basic fitness test and various strength and power measurements now in the US Army Combat Fitness Test (ACFT) and Occupational Physical Assessment Test (OPAT) for combat arms soldiers specifically [65]. Assessing strength in the military setting is complicated by the requirement for testing equipment, with few studies assessing this attribute. The results of the study by Kodesh et al. [30], who found no relationship between 10 m sprint time, drop jump, or countermovement jump and injury risk, may be skewed by the environment, where soldiers were attending a combat fitness instructors’ course. This may have led to those who were more athletically capable nominating themselves for this course and being included in the study. The single leg, triple hop distance did differentiate between female soldiers who were and were not injured [30], however, and so this warrants further investigation in future studies. Knapik et al. [28] found no association between any measure of upper or lower body strength or lower body power and injury risk in female soldiers during basic training. At this stage, measures of strength, speed and power only have minimal evidence of being a risk factor for injury in female soldiers.

### Flexibility

Flexibility was only assessed as an injury risk factor in one study [28], which found no relationship between flexibility and injury risk in female soldiers. A previous review has questioned the relevance of flexibility, due to its lack of correlation to military specific tasks [64]. Flexibility therefore does not appear to be associated with injury risk in female soldiers.

### Aerobic fitness

Aerobic fitness appears to be clearly related to injury risk in female soldiers, with all but one of the 11 included studies which reported on aerobic fitness showing significant associations between slow run times of any distance measured and injury risk, at all stages of a female soldier’s career. The importance of aerobic fitness is further highlighted by its strong correlation with a high number of military specific tasks [64]. The positive benefits of fitness appear to also negate the otherwise detrimental effects of a high BMI [27] or high percentage body fat [31]. The study by Rappole et al. [1], which did not find a significant association between injury risk and 2-mile run times, did not report run times of those who were and were not injured, making determination of the levels of fitness of each cohort difficult. The average run times in the overall combined cohort ranged from 17.87 to 18.31 min, slower than the 17 min cut off time used as a reference in the studies by both Grier et al., [35] and Roy [2]. Additionally, it may be that serving soldiers in that study possessed adequate fitness to protect against injury and that a ceiling effect may have been reached [63], or that the method of injury data capture might have only captured more severe injuries for which a soldier sought care [66]. It has been proposed that lower aerobic fitness, as opposed to sex/gender, is responsible for injury risk—in general, females have lower levels of aerobic fitness [67].

Some authors have advised against using a run time cut off for entry into military service prior to basic training, as there will be some who have slow run times who do not get injured and an injury does not necessarily mean an individual will not make it through basic training [25]. However, categorizing individuals by run time may serve to highlight to physical training instructors those who would be best targeted by ability-based training [68, 69], to avoid an excessive intensity of training and concurrent increased injury risk. The results suggest that low levels of aerobic fitness are a risk factor for injury in female soldiers.

### Movement assessments

Only two included studies assessed movement, via the FMS [30], and a Y Balance assessment [33], as potential risk factors for injury in female soldiers. The lack of observed association between FMS scores and injury risk may be due to the training location in which the assessment was conducted. As the authors [30] discussed, those female soldiers assessed were attending a combat fitness instructors’ course, and those who are more prone to injury may not have enrolled in this course. It should be noted that those who scored a zero in one or more movement assessments were more commonly injured during the course. Despite the composite score of the FMS as an injury prediction tool being more generally of questionable value [70], it has been found to be associated with injury risk within male military populations, albeit with a small magnitude of association [71]. Individual elements of the FMS may be more useful than the overall score, with those who scored a zero in one or more movement assessments more commonly injured during the combat fitness instructors course in the study by Kodesh et al. [30], and the pain provocation tests reported to predict injury in male US Army rangers [72]. The utility of the FMS, whether it be based on individual movements, pain clearing assessments or overall scores, remains unclear in female soldiers, due to both minimal research and low numbers of female participants.

Roy et al. [33] found that those female soldiers with poorer single leg balance ability as measured by the Y balance composite score were more prone to injury than those with better balance scores. In a similar manner, deficits in the Y balance assessment have been shown to be associated with patellofemoral pain in male military recruits [73], and in a multitude of sporting contexts [74, 75]. Its value in injury prediction for female soldiers warrants further investigation. Only minimal evidence exists for scores on movement assessments to be associated with injury risk in female soldiers.

### Limitations

Across the studies included in this review, there was wide variation in the definition of an injury, and this may have led to more minor injuries not being captured. The self-reporting of injuries is also problematic, as individuals may be reluctant to disclose them, fearing it may affect their entry to military service or their ongoing training. The focus of general injuries within this review may have missed risk factors for specific injuries. This may mean that some of the risk factors discussed here may or may not be risk factors for specific injuries. There were a variety of levels assessed for each risk factor, across the different studies, which made direct comparisons problematic. Entry to military service is also governed by fitness and medical standards, which may create a basement effect and exclude extremes of some measures (e.g., fitness, BMI, height, age) for many of the risk factors included. This may have led to a narrowing of the window for comparison for many measures and therefore may mean the findings of the review are not applicable to females outside of military service.

## Conclusion

Smoking, a lack of deployment history, heavy occupational tasks on deployment, low aerobic fitness and poor push-up performance are associated with increases in injury risk in female soldiers. Being deployed on operations may be protective of injury for female personnel, due to ensuring personnel are sufficiently medically fit for deployment and providing opportunity for personnel to conduct self-paced physical training. Age, sit-up ability, BMI, bodyweight, body fat percentage and height appear to have minimal or inconsistent associations with injury risk in female soldiers, while there also appears to be minimal evidence at this stage for movement assessments such as the FMS being predictors of injury risk in female soldiers. Strategies to improve aerobic fitness and upper limb endurance, reduce smoking, and optimize rehabilitation of injuries and risk management of heavy occupational tasks need to be developed to reduce injury risks for female soldiers.

## Data Availability

Not applicable.

## Abbreviations

ACFT: Army Combat Fitness Test
AIT: Advanced Individual Training
APFT: Army Physical Fitness Test
BMI: Body Mass Index
CASP: Critical Appraisal Skills Program
FMS: Functional Movement Screen
HR: Hazard Ratio
IDF: Israeli Defence Force
IRR: Incident Rate Ratio
OPAT: Occupational Physical Assessment Test
OR: Odds Ratio
PT: Physical Training
RR: Relative Risk
US: United States
